# Correlation between Ionospheric Spatial Decorrelation and Space Weather Intensity for Safety-Critical Differential GNSS Systems

**DOI:** 10.3390/s19092127

**Published:** 2019-05-08

**Authors:** Jinsil Lee, Jiyun Lee

**Affiliations:** Department of Aerospace Engineering, Korea Advanced Institute of Science and Technology, 291 Daehak-Ro, Daejeon 305-701, Korea; js515@kaist.ac.kr

**Keywords:** GNSS, ionospheric spatial decorrelation, space weather intensity, correlation, safety-critical navigation system

## Abstract

An ionospheric spatial decorrelation is one of the most dominant error factors that affects the availability of safety-critical differential global navigation satellite systems (DGNSS). This is because systems apply significant conservatism on the error source when ensuring navigation safety due to its unpredictable error characteristic. This paper investigates a correlation between GNSS-derived ionospheric spatial decorrelation and space weather intensity. The understanding of the correlation has significant advantages when modeling residual ionospheric errors without being overly pessimistic by exploiting external sources of space weather information. An ionospheric spatial decorrelation is quantified with a parameter of spatial gradient, which is an ionosphere total electron content (TEC) difference per unit distance of ionospheric pierce point (IPP). We used all pairs of stations from dense GNSS networks in the conterminous United States (CONUS) that provide an IPP separation distance of less than 100 km to obtain spatial gradient measurements under both ionospherically quiet and active conditions. Since the correlation results would be applied to safety-critical navigation applications, special attention was paid by taking into consideration all non-Gaussian tails of a spatial gradient distribution when determining spatial gradient statistics. The statistics were compared with space weather indices which are disturbance storm time (Dst) index and interplanetary magnetic field (IMF) Bz index. As a result, the ionospheric spatial decorrelation showed a significant positive correlation with both indices, especially under active ionospheric conditions. Under quiet conditions, it showed positive correlation slightly weaker than those under active conditions, and the IMF Bz showed preceding response to the spatial gradient statistics revealing the potential applicability for predicting the spatial decorrelation conditions.

## 1. Introduction

Global navigation satellite system (GNSS)-based safety-critical navigation systems are supporting world-wide aircraft operations while meeting the strict aviation requirements for various phases of flight from the en-route to the precision approach [1,2,3]. Safety-critical navigation systems guarantee that the aircraft navigation error does not exceed the pre-defined alert limit to an extremely high probability, which is defined by the system integrity requirement [1]. Currently, there are two standardized systems from the International Civil Aviation Organization (ICAO) Standards and Recommended Practices (SARPS) [1]: the ground based augmentation system (GBAS) [4] and the satellite based augmentation system (SBAS) [5]. Both of these systems achieve the aircraft precision approach with vertical guidance meeting the integrity requirement. Both systems rely upon monitoring GNSS signals by the precisely known positions of ground receivers, and broadcast range error corrections to users by exploiting the differential GNSS (DGNSS) technique. More importantly, integrity parameters accompany the corrections, providing information about the uncertainty of the corrections, as the corrections generated by ground systems cannot entirely capture the range error that an aircraft is experiencing.

The ionosphere is the most challenging error source when quantifying the uncertainty of the corrections due to its variable and unpredictable delay error characteristics in GNSS signals [6]. In GBAS and SBAS, which exploit ground receivers to correct the range error, a spatially decorrelated ionosphere results in residual ionospheric delay errors after applying the corrections. The ionospheric spatial decorrelation is usually quantified by a parameter of spatial gradient, which is defined as a difference in total electron content (TEC) or ionosphere delay error (which is proportional to TEC) per unit distance of the ionosphere pierce point (IPP). It was estimated that the ionosphere spatial gradient ranges from less than 0.01 TEC unit (TECU)/km under a quiet ionospheric condition to as large as 2.54 TECU/km observed during the storm event on 23 October 2013 [7,8].

The degree of ionospheric spatial decorrelation experienced by aircraft varies depending on ionospheric conditions. Without a method to distinguish different ionospheric spatial decorrelation conditions, the system should assume the worst ionospheric spatial decorrelation models when protecting user position error. Aviation communities have conducted extensive analyses and development for the past 30 years on an integrity algorithm against the residual ionospheric error uncertainty to protect aircraft navigation safety. GBAS determines a conservative standard deviation, denoted as σ_vertical ionospheric gradient (vig)_, of the ionospheric spatial gradient using historical datasets, and broadcasts it to a user within an airport using Very High Frequency (VHF) data broadcast (VDB) to bound correction uncertainty against residual ionospheric error under nominal ionospheric conditions [7,9,10]. Users always assume the pre-defined σ_vig_ when computing the nominal user position error bound. Furthermore, even though the σ_vig_ parameter overbounds various nominal ionospheric conditions from quiet to active events during most operational times, there is a possibility that the σ_vig_ parameter does not overbound the actual error due to the excessive ionospheric spatial gradient [11]. In addition to the σ_vig_ parameter, GBAS applies an upper bound of the spatial gradient threat model which was constructed using the worst-case spatial gradients observed from several severe ionospheric storm events to simulate the system availability against the potential threat conditions [8,9,12,13].

Unlike GBAS which monitors GNSS signals by ground receivers installed at each airport, the SBAS system monitors GNSS signals via the network of ground stations over the coverage area. The ionospheric range error and uncertainty information are then computed at the SBAS master station for each ionosphere grid point (IGP) and they are broadcast on the SBAS signal via a geostationary orbiting (GEO) satellite. As in GBAS, no detailed knowledge about the current ionospheric spatial decorrelation condition causes the SBAS ionospheric correction uncertainty or grid ionospheric vertical errors (GIVE) to be conservative by assuming the worst-case condition. This assumption was made in several terms used to determine GIVE. These include a sigma that specifies the nominal ionospheric decorrelation, an inflation factor that the nominal decorrelation sigma must be multiplied by to protect against all the errors for all ionospheric conditions, and an undersampled uncertainty term that represents a worst-case threat that the system would encounter in an undersampled geometry [14]. Of special note, the conservatism on the undersampled uncertainty term comes from an assumption that the worst-storm ever seen is about to influence its delay computation at all times [14,15].

As conservative as the parameters are, the reviewed methods still yield the systems operating in the conterminous United States (CONUS) region sufficient for meeting the current requirements which are Category (CAT)-I service for GBAS and localizer precision with vertical guidance (LPV) 200 for SBAS which guide aircraft down to 200 ft of the ground [16]. Nevertheless, both systems are actively working on improving system availability. GBAS is working on developing auto-land capability (defined by CAT-II/III service requirements) to guide an aircraft to the runway surface in all weather and visibility conditions [17], and SBAS is expanding its coverage for LPV-200 service [15]. Therefore, the model parameters must not be too conservative for system availability, but must safely bound true ionospheric behavior in order to assure integrity. We, therefore, require a method that will accurately describe the ionospheric behavior without being overly pessimistic. 

In response to this, this study investigates the feasibility of utilizing space weather information for GNSS-based safety-critical navigation systems based on the well-known fact that the ionosphere is coupled to the space weather activity [18,19,20,21]. Space weather (or geomagnetic) indices are provided by many organizations including the Space Weather Prediction Center (SWPC) from the National Oceanic and Atmospheric Administration (NOAA) [22] and the National Aeronautics and Space Administration (NASA) Space Weather Laboratory [23]. In addition, space weather prediction technologies have been significantly advanced and matured in recent years [22,24]. If the level of ionospheric spatial decorrelation is understood with reliable and measurable space weather indices in real-time or in advance, it can be utilized to describe the ionospheric spatial decorrelation without being overly conservative.

Previously, there were studies for the relationship between space weather and ionosphere. Mendillo et al. [19] found that ionospheric TEC enhancements coincided with increases of space weather activity. Balan and Rao [18] showed a correlation between space weather intensity with ionospheric activity strength by examining both low- and mid-latitude TEC and peak electron density data. Based on the relationship, GBAS and SBAS communities have utilized space weather indices to help target days on which anomalous or nominal behavior was likely to have occurred when developing the ionospheric models in the studies mentioned above [7,8,25]. However, due to a lack of information on the relationship between space weather indices and the ionospheric spatial gradient, which the safety-critical navigation systems specifically require, space weather indices could not be used to a broader extent for reducing the conservatism on the ionospheric spatial decorrelation models.

This study focuses on the relationship between the ionospheric spatial decorrelation and space weather indices which is an essential understanding for GNSS-based safety-critical systems. In our previous study, we have shown that the worst-case spatial gradient threat points observed from several ionospheric storm days are highly correlated with the disturbance storm time (Dst) index [26]. However, the study was limited to only an extreme ionospheric storm condition, and the relationship was derived using only several tens of spatial gradient threat points due to limited data observed during storm days. This paper uses all pairs of stations from dense GNSS networks in the conterminous United States (CONUS) region that provide an IPP separation distance less than 100 km to obtain spatial gradient measurements under both ionospherically quiet and active conditions. The statistics are then compared with space weather indices, disturbance storm time (Dst) index, and the interplanetary magnetic field (IMF) Bz, for the correlation study to the ionosphere. In addition, a case study is addressed discussing an example application of the developed relationship to GBAS which potentially reduces conservatism without modifying a core system architecture.

This paper is organized as follows: The dataset used for the correlation study is described in Section 2. Methods and algorithms for estimating TEC gradients in the CONUS region are discussed in Section 3. Section 4 presents the Gaussian probability density function (pdf) overbounding technique which is a key procedure for ensuring system integrity when modeling the navigation error. Section 5 shows the results of correlation analysis between statistics of TEC gradients and space weather indices. The discussion and conclusion are given in Section 6.

## 2. Dataset Used for the Correlation Study

### 2.1. Data Set

The analysis described in this paper was carried out using data from 9 consecutive days in 2004 (one of the years of active solar activity from the 11-year solar cycle) and 9 consecutive days in 2010 (one of the years of quiet solar activity from the 11-year solar cycle) to include all ionospherically quiet and active days that were not classified as ionospheric storm days to take into account overall ionospheric behavior. Table 1 shows two datasets (Dataset 1 and Dataset 2) representing active and quiet ionospheric conditions, respectively. The days were selected by considering storm classes as defined in [27]. The 5 storm classes are weak (−30 nT ≥ Dst_min_ > −50 nT), moderate (−50 nT ≥ Dst_min_ > −100 nT), strong (−100 nT ≥ Dst_min_ > −200 nT), severe (−200 nT ≥ Dst_min_ > −350 nT), and great (Dst_min_ ≤ −350 nT). It is shown that Dataset 1 covers various ionospheric storm classes from weak to strong, and Dataset 2 includes days which are classified as a quiet condition. Choosing consecutive days for the correlation study increased the reliability of the TEC gradient estimation against a long-term variation of data quality of stations.

### 2.2. CORS Data for TEC Gradient Estimation

The ionospheric delay, I (in meters), is proportional to TEC as shown in Equation (1), where K is a constant equal to 40.3 m^3^·s^−^^2^ and f is a carrier frequency of a signal (Hz) [3]. Using this relation, GNSS networks equipping dual-frequency GNSS receivers are enabled to estimate TEC gradients experienced between receivers for a short baseline distance of several tens of kilometers [28].

(1) $$I=\frac{K}{f_{}^{2}}\mathrm{TEC}$$

**Figure 1:**
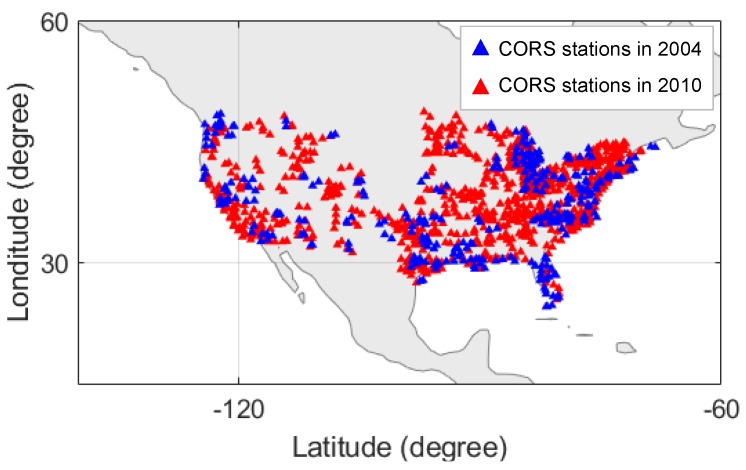
Continuously operating reference stations (CORS) stations in 2004 and 2010 used for the analysis in conterminous United States (CONUS) region.

The continuously operating reference stations (CORS) are a publicly available network of GNSS reference stations that provide GNSS data consisting of carrier phase and code range measurements throughout the U.S. and several other countries. The CORS network basically provides measurements of global positioning system (GPS) which is one of the GNSSs developed by the United States, but the number of stations that track Russia’s GLONASS measurements is also increasing. Given the large number of stations that provide GPS measurements, this study obtained L1 ($f_{L1}$ at 1575.42 MHz) and L2 ($f_{L2}$ at 1227.60 MHz) dual frequency GPS measurements from the CORS installed over the CONUS region with more than 400 stations in 2004 and more than 1000 stations in 2010 [29]. Figure 1 shows the available CORS stations in 2004 and 2010 used for the study in the CONUS region. Data with a 30-s sampling rate was used from all available stations. Using CORS data, precise TEC measurements are generated using the long term ionospheric anomaly monitor (LTIAM) software [25], and the estimation algorithm of the software will be discussed in Section 3.

### 2.3. Space Weather Indices for the Correlation Study

This study focuses on the relationship between statistics of ionospheric spatial decorrelation and corresponding space weather indices, but ultimately it is hoped that the relationship would potentially apply to safety-critical applications for improving system availability as introduced in the introduction section. Thus, it is important to select a suitable space weather index (or indices) considering accessibility, fast update rate, and availability of predicted data (in the case of predicting the ionospheric decorrelation conditions in advance), among other factors. Many organizations provide space weather indices in the form of ‘real time’ and/or ‘prediction’ values including the Dst index, planetary K (Kp), and IMF Bz, which are known to be correlated with ionospheric activity. 

A time resolution (or update rate) of real-time space weather data is considered as an essential factor for the correlation study. Among space weather indices, the Dst index and IMF Bz index provide an update rate of less than 1 h for real-time data, while the update rate of the Kp index is 3 h. The hourly Dst index is provided from the World Data Center for Geomagnetism, WDC-Kyoto, Japan [30], whereas 1-min resolution IMF Bz data were supplied by the WIND spacecraft [31], and provided by the NASA Goddard Space Flight Center available at [32].

The availability of the predicted value and the lead time of prediction are additionally considered when addressing the prediction of ionospheric spatial decorrelation conditions in advance in safety-critical applications. The lead time indicates the length of time between the broadcast of predicted data and the period for which the prediction is intended to be valid. In the case of the Dst index, predicted data is provided from the University of Colorado at Boulder [24] whose lead time and update rate are 50 min and 10 min, respectively. Thus, we selected the Dst data for the study considering the data update rate as well as availability for predicted data. Real-time Dst values provided by WDC-Kyoto are typically revised after a few months and replaced with ‘provisional’ Dst. After about two years, the ‘provisional’ Dst is replaced with a ‘final’ Dst. In this study, we applied the ‘final Dst’ for the correlation study. 

In addition, the IMF Bz index is also used for the study, although its predicted data is not provided. Prior studies have revealed that IMF Bz exhibits a faster response than Dst providing an explanation of the delays observed for these geomagnetic activity indices [33,34]. This study investigates if this characteristic could possibly be used in predicting the ionospheric spatial decorrelation conditions in advance.

## 3. TEC Gradient Estimation in the CONUS Region

Precise TEC estimates were generated using LTIAM software developed to monitor ionospheric events continuously [25]. The software utilizes GNSS dual frequency carrier (ϕ) and code (ρ) measurements to accurately estimate TEC based on the fact that the carrier measurement provides a range with much lower noise level (ε) compared with the code measurements, $\varepsilon_{\phi }\ll \varepsilon_{\rho }$, but contains unknown integer ambiguity numbers ($N_{L1}$,$N_{L2}$) not included in the code measurement. The LTIAM software computes the slant TEC based on the following steps:

First, the slant TEC estimates using carrier and code measurements are computed for each station i and satellite k pair, respectively, as shown in Equations (2) and (3), where K is a constant equal to 40.3 m^3^·s^−^^2^, γ is the combination of the carrier frequency of the signals (Hz) as $1/f_{L2}^{2}-1/f_{L1}^{2}$, and λ is the wavelength of the GNSS carrier signal.

(2) $${\mathrm{TEC}}_{\phi \_meas}{{}_{,}}_{i}{}^{k}=\frac{\phi_{L1}-\phi_{L2}}{K\gamma }={\mathrm{TEC}}_{i}{}^{k}-\frac{c}{K\gamma }(IFB_{i}+\tau^{k}{}_{gd})+\varepsilon_{\phi }+\frac{\lambda_{L1}N_{L1}-\lambda_{L2}N_{L2}}{K\gamma }$$

(3) $${\mathrm{TEC}}_{\rho \_meas}{{}_{,}}_{i}{}^{k}=\frac{\rho_{L2}-\rho_{L1}}{K\gamma }={\mathrm{TEC}}_{i}{}^{k}+\frac{c}{K\gamma }(IFB_{i}+\tau^{k}{}_{gd})+\varepsilon_{\rho }$$

The two TEC estimates include common terms which are a true TEC that we want to extract ultimately (${\mathrm{TEC}}_{i}{}^{k}$), and the inter-frequency hardware biases on the receiver ($IFB_{i}$) and satellite ($\tau^{k}{}_{gd}$) where c is the speed of light. The integer ambiguity numbers, as well as remaining noise, are additionally contained in ${\mathrm{TEC}}_{\phi \_meas}{{}_{,}}_{i}{}^{k}$. Utilizing the characteristics that the remaining error $\varepsilon_{\phi }$ included in the ${\mathrm{TEC}}_{\phi \_meas}{{}_{,}}_{i}{}^{k}$ is negligible, and the $\varepsilon_{\rho }$ included in the ${\mathrm{TEC}}_{\rho \_meas}{{}_{,}}_{i}{}^{k}$ is assumed to follow a zero-mean Gaussian distribution, the last term which includes the integer ambiguities in Equation (2) is estimated utilizing Equation (3) as shown in the next paragraph.

Second, a leveling is performed by computing the level parameter, $Level_{i}^{k}$, in order to remove the integer ambiguity numbers as shown in Equation (2). $Level_{i}^{k}$ is computed for each continuous arc by averaging the difference between the ${\mathrm{TEC}}_{\phi \_meas}{{}_{,}}_{i}{}^{k}$ and ${\mathrm{TEC}}_{\rho \_meas}{{}_{,}}_{i}{}^{k}$ over the epoch by applying a satellite elevation (el) dependent weighting as in Equation (4) where N is the number of data points within a continuous arc.

(4) $$Level_{i}^{k}=\frac{\sum_{n=1}^{N}\left(TEC_{\rho \_meas,}{}_{i}^{k}(t_{n})-TEC_{\phi \_meas,}{}_{i}^{k}(t_{n})\right)\sin^{2}el_{t_{n}}}{\sum_{n=1}^{N}\sin^{2}el_{t_{n}}}$$

The leveled carrier-derived TEC measurement ($TEC_{\phi \_leveled}{{}_{,}}_{i}^{k}$) is then computed by adding $Level_{i}^{k}$ to the ${\mathrm{TEC}}_{\phi \_meas}{{}_{,}}_{i}{}^{k}$ as in (5).

(5) $$TEC_{\phi \_leveled}{{}_{,}}_{i}^{k}=TEC_{\phi \_meas}{{}_{,}}_{i}^{k}+Level_{i}^{k}=TEC_{i}^{k}+\frac{c(IFB_{i}+\tau_{gd}^{k})}{K\gamma }$$

Third, the inter-frequency bias terms ($IFB_{i}$, and $\tau_{gd}^{k}$) are estimated. LTIAM applies estimates of $\tau_{gd}$ provided by the International GNSS Service (IGS) to remove the satellite hardware bias, and search for $IFB_{i}$ which minimizes the cumulative vertical TEC standard deviation to the mean on a given day [35]. After applying the hardware biases, $TEC_{i}^{k}$, which is used for the gradient estimation, remains.

Finally, $TEC_{i}^{k}$ and $TEC_{j}^{k}$ are used to compute a vertical TEC gradient based on the station-pair method [7] between stations i and j observing satellite k. The slant TEC is converted to an equivalent vertical TEC (${\mathrm{TEC}}_{V}$) using a geometric mapping function. In this study, we applied the mapping function derived by approximating the ionosphere which originally stretches from a height of about 50 km to more than 1000 km above the surface of the Earth [36] with a thin-shell model. The thin-shell model treats the entire ionosphere to be a shell of finite thickness containing the condensed TEC located at 350 km [5]. The mapping function or an obliquity factor, $M(el_{i}{}^{k},h_{I})$, which is a function of satellite elevation and ionospheric model height is shown in Equation (6) where $R_{e}$ is the radius of the Earth, $h_{I}$ is the height of the ionospheric shell, and $el_{i}{}^{k}$ is the elevation angle of the line of sight between a station i and a satellite k. The difference of the ${\mathrm{TEC}}_{V}$ between stations i and j becomes a differential vertical TEC (${\mathrm{dTEC}}_{V,}{{}_{ij}}^{k}$) as shown in Equation (7). The vertical TEC gradient (${\mathrm{TEC}}_{V}{\mathrm{gradient}}_{ij}{}^{k}$) is then computed by dividing ${\mathrm{dTEC}}_{V,}{{}_{ij}}^{k}$ with the distance $d_{ij}$ between ionospheric pierce points (IPP) (points where the line-of-sight (LOS) from two stations and the thin-shell ionospheric model intersect) as in Equation (8).

(6) $$M(el_{i}{}^{k},h_{I})={\left\{\cos\left[\sin^{-1}\left(\frac{R_{e}\cos(el_{i}^{k})}{R_{e}+h_{I}}\right)\right]\right\}}^{-1}$$

(7) $$\begin{array}{ll} {\mathrm{dTEC}}_{V,ij}{}^{k} & ={\mathrm{TEC}}_{V,i}{}^{k}-{\mathrm{TEC}}_{V,j}{}^{k}\\ & =M(el_{i}{}^{k},h_{I})\cdot {\mathrm{TEC}}_{i}{}^{k}-M(el_{j}{}^{k},h_{I})\cdot {\mathrm{TEC}}_{j}{}^{k} \end{array}$$

(8) $${\mathrm{TEC}}_{V}\;{\mathrm{gradient}}_{ij}{}^{k}=\frac{{\mathrm{dTEC}}_{V,}{{}_{ij}}^{k}}{d_{ij}}$$

**Figure 2:**
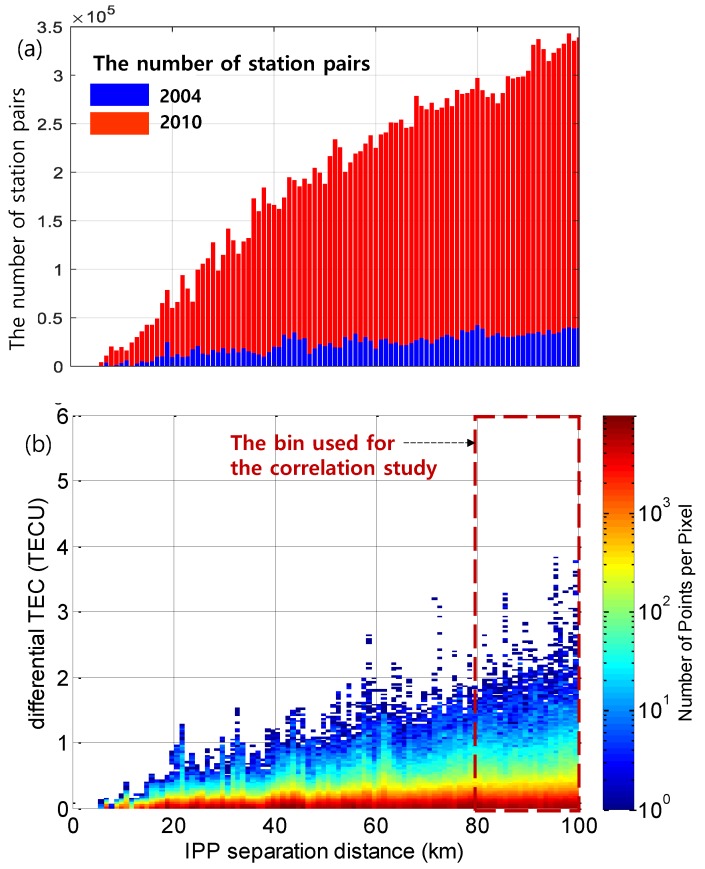
A number of station pairs used to derive vertical total electron content (TEC) statistics (**a**) and a differential TEC histogram on 26 July 2004 (a disturbed day) as a function of ionospheric pierce point (IPP) separation distances (**b**).

Based on the reviewed algorithm, differential TEC as a function of IPP separation distance derived using data on 26 July 2004 is shown in Figure 2b. The numbers of station pairs used for statistical analysis in 2004 and 2010 are also shown in Figure 2a along with the differential TEC, revealing a significant increase in the number of CORS stations. In Figure 2b, horizontal axes divide the IPP separation distance, and vertical axes divide differential vertical TEC into bins, the color of which corresponds to the number of performed measurements. Herein, TEC_V_ gradients were estimated using station pairs with IPP distances of less than 100 km, mimicking the situation when an aircraft receives DGNSS corrections at an airport. The histogram above shows a linear and smooth dependence of differential TEC on IPP separation for the whole range of distances. For the correlation study, the bin from 80 km to 100 km was used to derive the reliable statistics considering the number of data points as well as the estimate uncertainty due to the remaining bias (e.g., the receiver inter frequency bias calibration error or the carrier-phase leveling error). The same amount of bias divided by a shorter distance would magnify the bias effect on vertical gradient estimates, and this effect is shown in Section 4.

## 4. Gaussian PDF Overbounding Technique

GNSS-based safety-critical navigation systems including GBAS and SBAS commonly model the ionospheric errors as a Gaussian distribution because of its simplicity for defining the error distribution with only two parameters (its mean and standard deviation) [37]. In this context, a standard deviation determined without thoroughly considering non-Gaussian tails may threaten the users. Thus, the Gaussian pdf overbounding technique, which has been utilized for the certification of GNSS-based aviation systems to ensure safety from navigation errors, is vital for handling errors to protect users even from low-probability threats. This method overbounds distributions reliably and conservatively by including non-Gaussian tails to achieve the required level of integrity. In this study, it is observed that the TEC gradient distribution has thick Gaussian tails so that the use of this method is required due to the subsequent application of analysis results in safety-critical applications.

**Figure 3:**
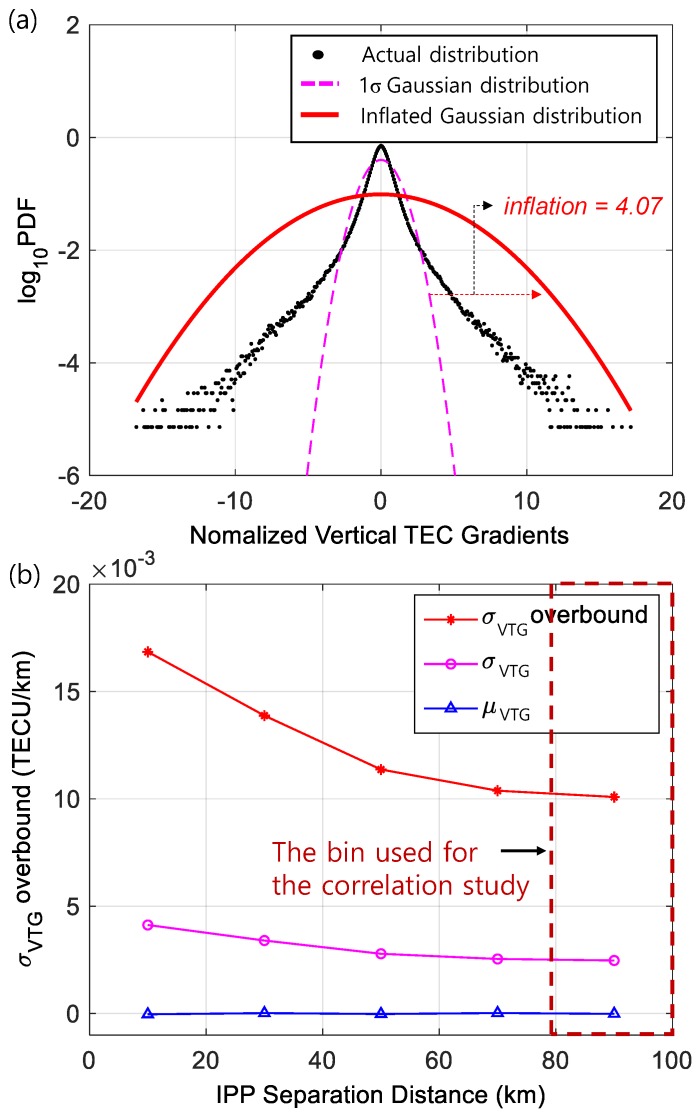
Results of Gaussian probability density function (pdf) overbounding using data of 26 July 2004. (**a**) Normalized vertical TEC gradient distribution with overbounded Gaussian distribution by applying an inflation factor, and (**b**) means, standard deviations, and overbounded standard deviations of vertical TEC gradients for each bin.

Figure 3a shows a pdf for normalized vertical TEC gradients in log scale. The normalized vertical TEC gradients are computed by removing their means and dividing them by their standard deviations, which were computed separately for each bin as illustrated in Figure 3b. Bin sizes for 2004 and 2010 were chosen as 20 km and 10 km, respectively, considering the number of data points required for reliable statistics. Normalized vertical TEC gradients displayed in Figure 3a show that the distribution has non-Gaussian (thick) tails. Considering that the ionospheric noise is modeled as a Gaussian distribution that is commonly used in GNSS-based aviation systems, the non-Gaussian tails must be appropriately overbounded by a Gaussian distribution with an inflated standard deviation. Thus, the nominal sigma (1σ) of a zero-mean Gaussian distribution (the dashed curve) was inflated to cover the non-Gaussian tails of the actual distribution. The inflation factor (f) bounding the distribution to the level of a probability of 1 × 10^−5^ is determined to take into account worst-case TEC gradients, and the inflated distribution is also shown in the same figure (solid line). The ‘σ_VTG overbound_’ is then computed as |μ_VTG_| + f∙σ_VTG_ by applying an inflation factor, where μ_VTG_ is a mean and σ_VTG_ is a standard deviation of a vertical TEC gradient for each bin as shown in Figure 3b. As mentioned in Section 3, the σ_VTG overbound_ increases as the distance decreases because the remaining biases are divided by the short distances and magnify the effect of biases on the estimates. In this study, σ_VTG overbound_ from the last bin (the bin of 80–100 km in 2004, and the bin of 90–100 km in 2010), which contains the largest number of data points with the longest IPP separation distances, is used for the correlation study.

## 5. Results

### 5.1. Daily Variations of the σ_VTG_overbound_ and Space Weather Indices

The daily variations of the σ_VTG_overbound_ and space weather indices (Dst and IMF Bz) are compared under both ionospheric active (Dataset 1) and quiet conditions (Dataset 2). The time interval of the data used in the comparison was set to ‘one day’ to obtain a reliable statistic for the σ_VTG_overbound_ value by securing a sufficient amount of data. In this response, values of daily maximum space weather indices were compared with the σ_VTG_overbound_ variations, and hourly maximum space weather indices are also shown as a reference.

**Figure 4:**
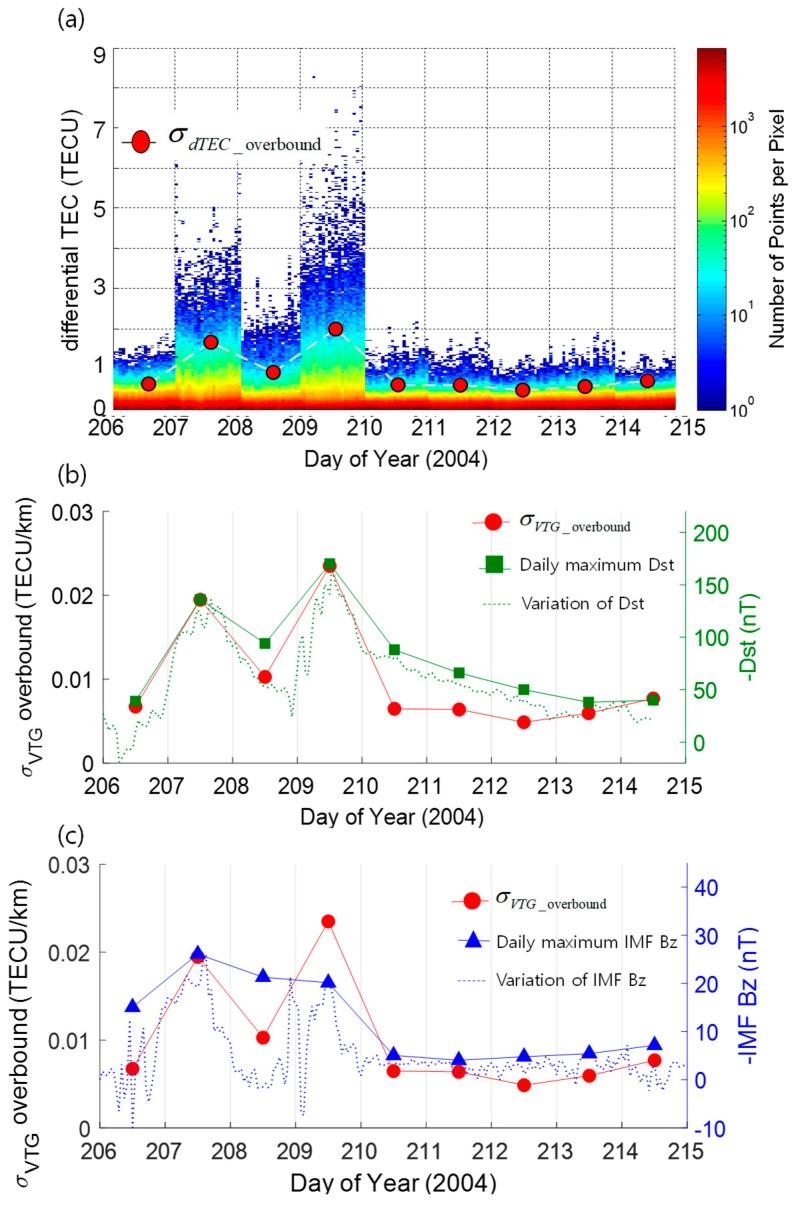
A series of a histogram of differential TEC in 2004 in (**a**), and variations of σ_VTG_overbound_ and corresponding daily and hourly maxima of negative disturbance storm time (Dst) (**b**), and negative interplanetary magnetic field (IMF) Bz (**c**), respectively.

Figure 4a shows a series of histograms of differential TECs extracted from the bin of IPP separation distance from 80 km to 100 km. The color of the histogram shows the number of points per pixel (the same description as in Figure 2). On top of the series of nine histograms, overbounded standard deviations of differential TEC values for each bin are overlapped with red dots to visualize the differential TEC variations. The overbounded standard deviations of differential TEC values were determined by multiplying the σ_VTG_overbound_ with the median value of the distance of the last bin. In Figure 4b,c, the daily variation of σ_VTG_overbound_ determined based on the method described in Section 4 under active ionospheric conditions is shown with the red dots. For comparison, the red dots are shown with the daily maximum values of space weather indices, −Dst index as squares in Figure 4b and −IMF Bz index as triangles in Figure 4c, which were determined from the maximum values of hourly space weather indices (plotted with dotted curves). Corresponding values are shown with the y-axis on the right. The negative sign in the axes of space weather indices is added for an easier understanding of the magnitude both for Dst and IMF Bz indices.

The observed daily variations of σ_VTG_overbound_ showed significant correlation with both Dst and IMF Bz indices. It is noteworthy that there is a limitation arising from analyzing the relationship by using the daily interval data. In the case of IMF Bz index and σ_VTG_overbound_ in Figure 4c, the hourly variations of IMF Bz show a significantly higher similarity with the variations of σ_VTG_overbound_ than the daily IMF Bz variations. This is a trade-off problem between the level of confidence on the determined statistics of vertical TEC gradients and the temporal precision on the correlation analysis. Since potential applications of this analysis are a safety-critical navigation system which requires a significant level of system integrity, this study limited the interval to ‘one-day’ to secure a sufficient amount of data.

**Figure 5:**
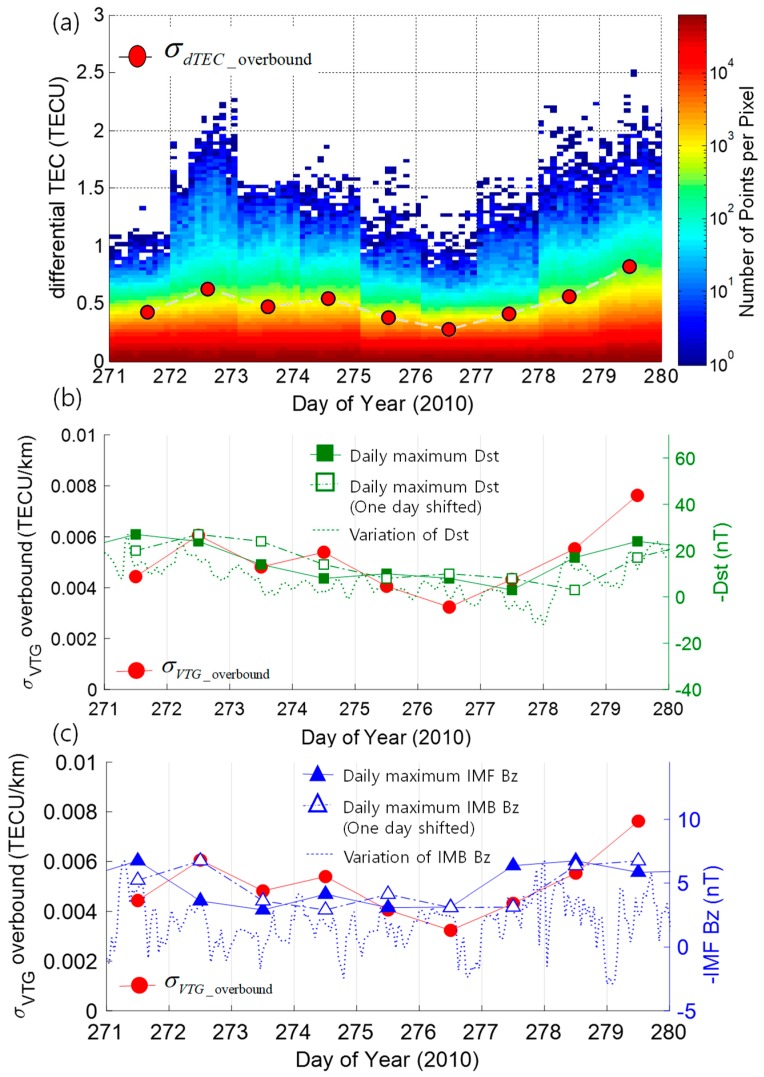
A series of differential TECs of differential TEC in 2010 (**a**), a comparison between σ_VTG_overbound_ and corresponding daily and hourly maxima of negative Dst (**b**), and negative interplanetary magnetic field (IMF) Bz (**c**), respectively. One day shifted negative Dst, and IMF Bz plots are also compared to the σ_VTG_overbound_.

The same procedure was repeated for Dataset 2 to examine the relationship shown above under ionospherically quiet conditions, revealing much smaller σ_VTG_overbound_ values, as shown in Figure 5. Each histogram was extracted from a bin of IPP separation distance from 90 km to 100 km. In this case, σ_VTG_overbound_ also exhibited a positive correlation but with less similarity compared to Dataset 1. In addition, its temporal correlation is slightly weaker than that of Dataset 1 (Figure 4). Notably, in our one-day-resolution analysis, the response of σ_VTG_overbound_ tended to be delayed by one day relative to those of both geomagnetic indices especially in case of IMF Bz index. IMF Bz values shifted by one day are plotted in Figure 5c to highlight the improved similarity in variations after considering the response delay. This finding coincides with the results of previous studies, and this will be discussed in Section 6 after quantifying the correlation of variations between σ_VTG_overbound_ and space weather indices in Section 5.2.

### 5.2. Correlation Study

**Figure 6:**
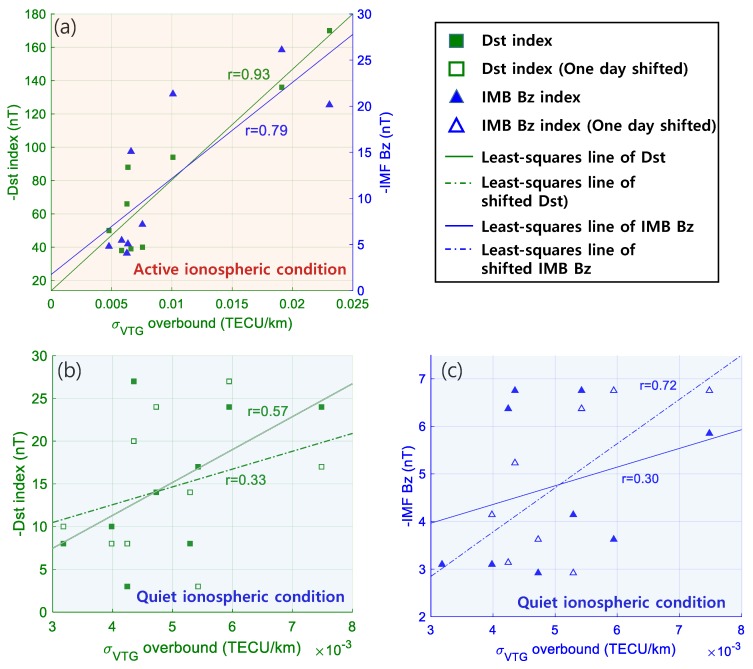
Pearson’s correlation coefficients determined for active ionospheric conditions using Dst and IMF Bz (**a**), and for quiet conditions using Dst index (**b**) and IMF Bz index (**c**).

Correlation analysis was conducted to quantify the obtained relationships, and Pearson’s correlation coefficients were determined. As expected, the correlation coefficient was considerably higher for active ionospheric conditions (0.93 with Dst index and 0.79 with IMF Bz index), confirming that TEC gradients were strongly correlated with space weather activity intensity as represented in Figure 6a. Because the correlation analysis was based on daily maxima, it exhibited some limitations, such as those set to a larger value of negative IMF Bz on day 208 compared to hourly variations on the same day (in Figure 4c). The correlation established for IMF Bz is expected to improve if the corresponding analysis is performed with improved temporal precision of data points. For ionospherically quiet conditions, the correlation coefficients were less than those in the active ionospheric case (as shown in Figure 6b,c). However, results showed a positive correlation with both indices. In particular, the correlation coefficient dramatically increased from 0.30 (for not shifted IMF Bz index) to 0.72 (for one-day-shifted IMF Bz) after considering the temporal delay in the case of the IMF Bz index. In the case of Dst, the tendency was not as dramatic, with the correlation coefficient slightly decreasing from 0.57 to 0.33 for 1-day-shifted Dst. This difference can be explained by the results of prior studies [33,34] and is discussed in the following section.

## 6. Discussion and Conclusions

This study examined the correlations of vertical TEC gradients with indices describing the intensity of space weather activity. Correlations obtained under active ionospheric conditions for both Dst and IMF Bz were higher than those determined under quiet conditions. Space weather events in Dataset 2 were much less pronounced (worst observed Dst of approximately −27 nT during our processed nine days) than those in Dataset 1 (worst observed Dst of approximately −170 nT during our processed nine days). The increase of the σ_VTG_overbound_ values was observed roughly one day after that of space weather indices, whereas the ionosphere was instantly impacted by the space weather activity in Dataset 1. The weaker correlation observed under quiet conditions was partially due to the delayed response of the TEC gradient statistics to space weather activity, as supported by previous studies on the relationship between time delay (between space weather activity and ionospheric storm occurrence) and the intensity of ionospheric activities at low- and mid-latitude stations [18]. It observed that time delays were inversely related to storm intensity based on data for more than 60 geomagnetic storms.

Another interesting observation is the decreased tendency of the Dst index in Dataset 2 to exhibit a time delay, as compared to that of IMF Bz, i.e., the time delay was clearly observed in the case of IMF Bz, being relatively unpronounced in the case of Dst. Prior studies have revealed that IMF Bz exhibits a faster response than Dst [33,34], providing an explanation of the delays observed for these space weather activity indices. 

**Figure 7:**
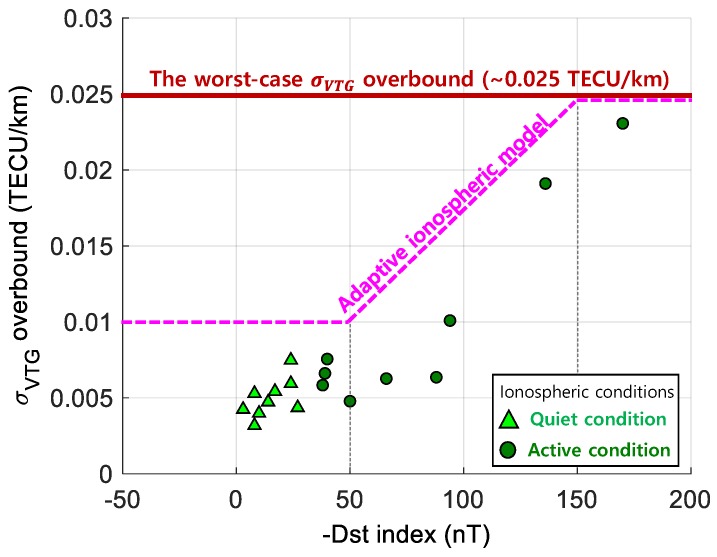
An example of the adaptive ionospheric model using the relationship between Dst and σ_VTG_overbound_.

These correlations would be applicable to safety-critical navigation systems. As a case study, Figure 7 shows an example application of the developed relationship to the local area augmentation system (LAAS), which is one of the GBAS operating in the CONUS region. Currently, LAAS is applying the worst-case σ_VTG_overbound_ of 0.025 TECU/km to an aircraft regardless of ionospheric conditions [7]. This could pose too much conservatism for the majority of the operation time. The solid red line in Figure 7 is the currently broadcast upper bound of a standard deviation of the vertical TEC gradients. We plotted a total of 18 σ_VTG_overbound_ points from both Dataset 1 and Dataset 2 as a function of Dst index. Triangle marks indicate the points observed from quiet ionospheric conditions, and circle marks represent the points observed from active ionospheric conditions. As discussed, the dependency or correlation of σ_VTG_overbound_ in active ionospheric conditions is higher with the Dst index than those of quiet ionospheric conditions. Based on the observed points, we discussed the potential possibility of reducing the conservatism with the adaptive ionospheric model, instead of using the worst-case upper bound model. The adaptive ionospheric model can be utilized to determine corresponding σ_VTG_overbound_ values by applying a real-time or a predicted Dst index while reducing conservatism. Even though we found that the correlation might be utilized to improve the availability of a safety-critical navigation system, it should be noted that great care must be taken in the practical application of these kinds of ionospheric models. Most importantly, safety verification should be conducted from factors such as the number of datasets used to develop a model or uncertainty of a real-time or predicted space weather index.

This study provides an understanding of the correlation between ionospheric spatial decorrelation and space weather activities. The results, combined with dramatically improving space weather technologies, could potentially provide the opportunity to foresee ionospheric impact under both nominal and ionospheric active conditions to broad applications which utilize DGNSS techniques. Further investigations are required to make this concept fully applicable to aviation systems. For instance, we limited the temporal resolution of TEC gradient statistics to ‘one day’ for the correlation study to ensure a sufficient amount of data. The increased number of station-satellite pairs obtained from multi-constellation GNSS satellites should enable the collection of a more substantial amount of data to reduce the time resolution and to improve the accuracy of analysis in terms of temporal correlation.

## Figures and Tables

**Table 1:** Dataset 1: Nine days in 2004 (active ionospheric condition) and Dataset 2: Nine days in 2010 (quiet ionospheric condition).

Dataset 1: Ionospherically Active Condition			Dataset 2: Ionospherically Quiet Condition		
Days in 2004: mm/dd (day of year)	Storm Class [27]	Minimum Dst (Disturbance Storm Time) (nT)	Days in 2010: mm/dd (day of year)	Storm Class [27]	Minimum Dst (nT)
07/24 (206)	weak	−39	09/28 (271)	quiet	−27
07/25 (207)	strong	−136	09/29 (272)		−24
07/26 (208)	moderate	−94	09/30 (273)		−14
07/27 (209)	strong	−170	10/1 (274)		−8
07/28 (210)	moderate	−88	10/2 (275)		−10
07/29 (211)	moderate	−66	10/3 (276)		−8
07/30 (212)	moderate	−50	10/4 (277)		−3
07/31 (213)	weak	−38	10/5 (278)		−17
08/01 (214)	weak	−40	10/6 (279)		−24
